# *mSphere*’s journey and future: a message from the new editor in chief

**DOI:** 10.1128/msphere.00337-25

**Published:** 2025-07-01

**Authors:** Ira J. Blader

**Affiliations:** 1 Biomedical Sciences and Pathobiology, Virginia-Maryland College of Veterinary Medicine 70732, Blacksburg, Virginia, USA

## EDITORIAL

Readers know that *mSphere* was launched in 2015 as a pan-microbial, multidisciplinary open-access journal that publishes high-quality, high-impact work across the microbial sciences. However, some may not know that *mSphere* came with a charge from its publisher, ASM: to publish rigorous and high-quality science with rapid peer-review times as well as to develop and conduct publishing experiments and trials focused on developing scientific publishing processes that are more equitable and less time-consuming for both authors and reviewers.

The previous paragraph paraphrases Dr. Michael Imperiale’s email to me ~11 years ago inviting me to join *mSphere* as a senior editor. As a mid-career faculty member, I was not really looking for extra “work” at that time, but Mike’s last point intrigued me, and I joined *mSphere*’s editorial team. Since that time, and under his guidance and leadership, *mSphere* has grown into exactly what he and ASM envisioned: It is now an established and leading microbial sciences journal that is not afraid to test out new ideas.

As I was considering applying for the *mSphere* editor in chief (EIC) position, I reflected on that conversation with Mike and thought about the vision and goals he set forth for the journal. Certainly, the scientific landscape has changed since then, as has microbiology/academic publishing. Probably the most impactful (at least for American scientists) is that politics have become a dominating force in science that affects not only how we staff, run, and fund our laboratories but also how we communicate our science. And it was that last point that compelled me to apply for the EIC position, as I felt that of the high-quality microbiology journals, *mSphere* was well positioned to adapt and change with the times, and I was most excited about this.

So, what does the future have in store for *mSphere* and its readers and reviewers? First and foremost, our core mission continues: publish high-quality, high-impact work across the microbial sciences spectrum, and we will strive to further decrease times to first and final decisions. This leads to the next obvious question: what will change and what are my goals? First, *mSphere* will become a more global journal by not only reaching out and encouraging submissions from outside of the United States but also by diversifying our reviewers and editors. This starts with our senior editors, as not only is Dr. Imperiale rotating off as EIC, but six of our original senior editors (Drs. Sarah D’Orazio, Paul Duprex, Trina McMahon, Aaron Mitchell, and Susannah Tringe) are doing so as well. Before introducing their replacements, I want to take a moment to thank each of them for their service. I cannot think of a more intelligent, dedicated, and unselfish group of scientists than those I have had the privilege of working with on *mSphere*’s Senior Editor Board. I’ve learned so much from each of them and will be forever grateful that our paths crossed.

Our new senior editors (Drs. Lark Coffey, Ryo Handa, Michael D. L. Johnson, Jo Parish, Rebecca Shapiro, and Garret Suen) are an equally impressive group, and I am excited about what they bring to the table. First off, this will be the first time that these individuals, who are mid-career faculty members with extensive experiences as editors and reviewers, will have the opportunity to serve as a senior editor. Thus, they will bring new perspectives to the journal. Second, three of the six new senior editors are not from the United States. Thus, I expect that they will bring fresh ideas and new approaches to *mSphere*. In addition, we have recruited our first-ever minireview editor, Dr. Bing Zhai.

Like scientific discovery itself, publishing is a team effort: authors submit papers, reviewers assess the work, editors convey assessments back to authors and make decisions based on these reports and authors’ responses, and the journal staff keeps peer review running smoothly and then publishes, posts, and promotes papers. At *mSphere*, our focus is ensuring that this is not only a fair and rigorous process but one that is also supportive and constructive. Our goal will be to streamline and balance the process by continuing to implement and update our structured peer review form/system, asking reviewers to focus on the work they have in front of them and not the paper they wished they had in front of them, expecting editors to judiciously call upon reviewers to reevaluate resubmitted papers, and partnering with Chronoshub to develop pain-free submission processes (https://asm.org/press-releases/2025/march/asm-launches-on-chronoshub-with-enhanced-article-s).

One challenge for authors has been understanding the roles and foci of ASM’s pan-microbial sciences journals (including *mSphere*) and how they are integrated and fit with other ASM journals. Probably the most common questions I get are “What makes *mSphere* different than the other ASM journals?” and “Is *mSphere* the right home for my paper?” My first answer, and one we task our reviewers and editors with, is to ask “Do you feel that your paper fits within the top half of the papers in your field?” because that is what we are focused on publishing. These could include papers that present novel findings but with limited mechanistic insight, proof-of-concept results for novel experimental approaches that can then be applied to a variety of biological problems, expanded and/or in-depth assessment of how microbial ecology and niches impact biodiversity and ecosystems, and the development of new and/or significantly refined clinical tests. If authors are unsure whether their manuscript might be a fit for *mSphere*, they are welcome to email the journal at msphere@asmusa.org pre-submission inquiries in the form of a manuscript outline or abstract, and the senior editors will provide feedback on whether the proposed manuscript is within our scope.

Finally, I want to highlight several new collections that we are planning. Our experiment with the mSphere of Influence articles showed us that our readers want to get to better know their colleagues and the people behind the science. In today’s world, I would argue that we need more of this, and thus, we are working on developing new article formats that enable us to meet and learn about each other. These will not be restricted to only lab principal investigators but rather all involved in the scientific process (e.g., trainees, policy developers, and funders).

I want to close by thanking you for supporting *mSphere* over the past 10 years. We are a community, and my primary mission will be to continue to build *mSphere* as the microbial sciences community’s journal. Replacing Dr. Imperiale as EIC is a daunting prospect, but one that I consider to be a humbling task and great honor. My “door” is always open to all for ideas, questions, or concerns about the journal. And while I want to hear about how we can improve, I also welcome hearing notes highlighting what *mSphere* is doing right. I look forward not only to hearing and meeting you but also to reading and learning about the amazing science you all do.

